# A head-to-toe makeover for classical sequencing-by-synthesis helps users to squeeze more out of each base

**DOI:** 10.1093/nsr/nwy013

**Published:** 2018-02-10

**Authors:** Jianbin Wang, Angela Wu

**Affiliations:** 1 School of Life Sciences, Tsinghua University, China; 2 Division of Life Science, Hong Kong University of Science and Technology, China; 3 Department of Chemical and Biological Engineering, Hong Kong University of Science and Technology, China

Today, many next-generation sequencing (NGS) methods, technologies and platforms have been developed that allow users to produce Tera base pairs of data in less than one day or ultra-long reads covering multiple genes. By far the most popular NGS approach to date is cyclic sequencing-by-synthesis (SBS), whereby each DNA template's complementary strand is synthesized by DNA polymerase and, as the new strand grows base by base, the identity of each incorporated dNTP is detected via different chemistries or modalities. Existing variations on this SBS approach each has its strengths, but also each comes with trade-offs: modalities with high accuracy often suffer from reduced sequencing speed or read length; others can achieve long read length but at the cost of accuracy. In their recent *Nature Biotechnology* publication [1], Chen and colleagues give the classical SBS method an innovative head-to-toe makeover that could mean yet another quantum leap in sequencing speed, accuracy, and cost reduction.

In their error-correction code (ECC) sequencing strategy, Chen *et al.* are able to maintain extremely high sequencing accuracy over a read length of up to 250 bp and, of those, 200 bp are completely error-free. First, they addressed the issue of signal detection sensitivity and degradation by using superior fluorogenic sequencing substrates—deoxynucleotides that are phospho-linked to Tokyo Green fluorophore—which leaves non-terminated 3′ ends upon incorporation into the nascent strand and cleavage of the fluorophore group by DNA polymerase. The Tokyo Green fluorophores themselves boast a higher performance than other fluorogenic dyes and allow greater detection sensitivity; the natural ends after base incorporation greatly reduce cycle phasing and pre-mature strand termination issues, overall improving read length while maintaining signal fidelity. A bonus benefit of using a single dye color is the reduced reagent cost.

**Figure 1.**
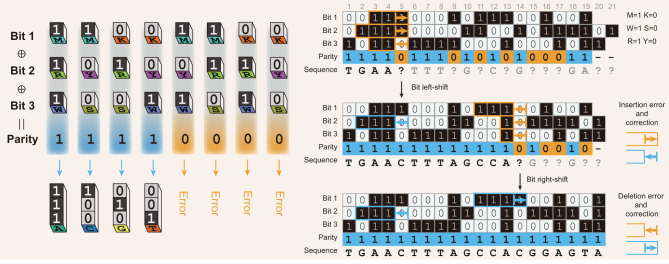
Information theory-based sequencing error correction. Left: Based on the two-base flow sequencing scheme (M—AC together; K—GT together; R—AG together; Y—CT together; W—AT together; S—CG together), a complete run-through of all two-base reaction mixtures can generate eight possible codewords, but only four are legitimate (with parity 1), while others indicate errors. Right: A simplified example of sequencing error correction using parity checking.

The second stroke of ingenuity is the use of dual-base flow rather than assessing the incorporation of one base each cycle, as is the convention. Specifically, their scheme flows in two bases in each cycle, and alternately introduces each dual-base combination in subsequent cycles. In each cycle of this so-called ‘degenerate sequencing' approach, the fluorescence gives a measure of the number of bases in that dual-base combination that was incorporated, without specifically denoting the base itself. An information communication model is then used to decode the base identity from the degenerate sequence data (Fig. 1). Using this method, each template is synthesized and sequenced multiple times with redundancy in each read, thereby effectively creating built-in double-checking of each base to further ensure accuracy. Interestingly, one could also imagine that a single round of dual-base flow degenerate sequencing, obtainable at double the speed of single-base flow SBS, could be sufficient for a rough alignment in certain applications such as copy number variation (CNV) profiling and RNA-seq.

All signs indicate that the trend of decreasing sequencing cost will continue but, when each base costs a fraction of a cent, what matters will no longer be the dollar value of each SBS cycle, but rather the amount of information represented. ECC sequencing essentially increases the amount of information generated with no compromise in speed and, in many cases, the resulting improved base-accuracy also saves the cost of having to sequence more deeply. This is where the true value-add of ECC sequencing is: it provides users with the flexibility to exchange speed for accuracy and vice versa, without sacrificing read length. An ultra-high-speed sequencer is arguably the missing piece that could bring NGS closer to point-of-care use, whilst an ultra-accurate sequencer could be the game-changer for many precision medicine applications. Either way, ECC is poised to disrupt the sequencing technology space and lead with new quality standards in NGS sequencing.
